# Direct Synthesis of Polyethylene Thermoplastic Elastomers Using Hybrid Bulky Acenaphthene-Based α-Diimine Ni(II) Catalysts

**DOI:** 10.3390/molecules28052266

**Published:** 2023-02-28

**Authors:** Hui Wang, Weiqing Lu, Mingmin Zou, Shengyu Dai

**Affiliations:** 1 School of Chemistry and Chemical Engineering, Hefei Normal University, Hefei 230061, China; 2 School of Chemical and Environmental Engineering, Anhui Polytechnic University, Wuhu 241000, China; 3 Information Materials and Intelligent Sensing Laboratory of Anhui Province, Institutes of Physical Science and Information Technology, Anhui University, Hefei 230601, China

**Keywords:** α-diimine nickel complexes, branched polyethylenes, ethylene polymerization, polyethylene thermoplastic elastomers

## Abstract

Recently, polyolefin thermoplastic elastomers can be obtained directly using ethylene as a single feedstock via α-diimine nickel-catalyzed ethylene chain walking polymerization. Here, a new range of bulky acenaphthene-based α-diimine nickel complexes with hybrid *o*-phenyl and -diarylmethyl anilines were constructed and applied to ethylene polymerization. All the nickel complexes under the activation of excess Et_2_AlCl exhibited good activity (level of 10^6^ g mol^−1^ h^−1^) and produced polyethylene with high molecular weight (75.6–352.4 kg/mol) as well as proper branching densities (55–77/1000C). All the branched polyethylenes obtained exhibited high strain (704–1097%) and moderate to high stress (7–25 MPa) at break values. Most interestingly, the polyethylene produced by the methoxy-substituted nickel complex exhibited significantly lower molecular weights and branching densities, as well as significantly poorer strain recovery values (48% vs. 78–80%) than those by the other two complexes under the same conditions.

## 1. Introduction

**Scheme 1:**
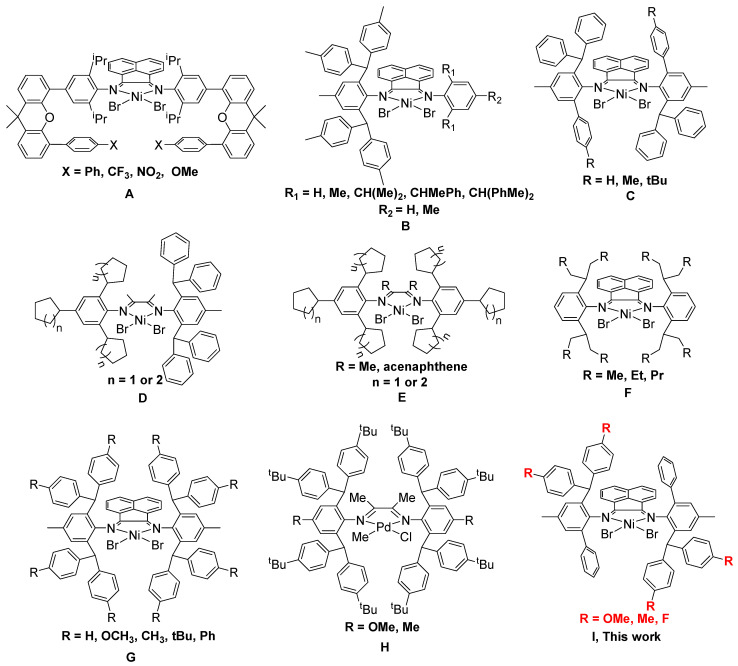
Bulky *α*-diimine Ni(II) and Pd(II) catalysts for the preparation of polyethylene TPEs in previous works (**A**–**H**) and our current work (**I**).

As a kind of high-performance polyolefin material, polyolefin thermoplastic elastomers (TPE) can be processed at high temperatures and exhibit rubbery properties at room temperature. Such polyolefin materials thus have the advantages of both rubber and plastic. They are widely used in the automotive industry as high-performance accessory materials and photovoltaic film fields [1,2]. Most polyolefin TPEs in the industry today are available through metallocene-catalyzed copolymerization of ethylene with α-olefins [3,4]. Recently, it is possible to prepare polyolefin TPEs directly by using ethylene as a single raw material via α-diimine nickel-catalyzed ethylene chain walking polymerization [5,6,7,8,9,10,11,12,13,14,15,16,17,18,19,20,21]. The direct preparation of polyolefin TPEs with only ethylene feedstock is extremely attractive and shows great application potential. For example, recently, Chen, Jian, Sun and our group have designed a series of novel unsymmetrical nickel α-diimine catalysts (Scheme 1A–D) to catalyze the polymerization of ethylene to obtain high-performance polyethylene TPEs [5,6,8,11,15,20,21]. Controlling the ratio of chain walking to chain growth by the reaction temperature and ethylene pressure to obtain polyethylene of a certain crystallinity is the key to preparing high-performance polyolefin TPEs. High molecular weight is one of the necessary requirements [17]. Moreover, some symmetrical bulky nickel α-diimine catalysts (Scheme 1E–G) have also been shown to catalyze the polymerization of ethylene to obtain polyethylene material with high elastic recovery [7,9,10]. The carbon spectrum analysis of the obtained polyethylene shows that the distribution of branching in these branched polyethylenes is random and methyl branching dominates. Different from α-diimine nickel catalysts, the corresponding α-diimine palladium catalysts tend to possess excessive chain-walking ability, producing fully amorphous polyethylene [22,23,24,25,26,27,28,29,30,31,32,33]. We have recently succeeded in suppressing chain walking in palladium-catalyzed ethylene polymerization using a bulky *o*-aryl substitution strategy [34,35]. By selecting suitable *o*-aryl substituents, the α-diimine palladium catalysts (Scheme 1H) can also catalyze the polymerization of ethylene to obtain the corresponding polyethylene TPEs [36]. What is more, polar functionalized polyethylene TPEs can also be prepared by co-polymerization of ethylene with polar monomers [36]. In this study, a series of new hybrid bulky acenaphthene-based α-diimine Ni(II) catalysts were synthesized and applied to prepare polyethylene TPEs with excellent recovery performance.

## 2. Results and Discussion

### 2.1. Synthesis and Characterization of α-Diimine Ni(II) Complexes

**Scheme 2:**
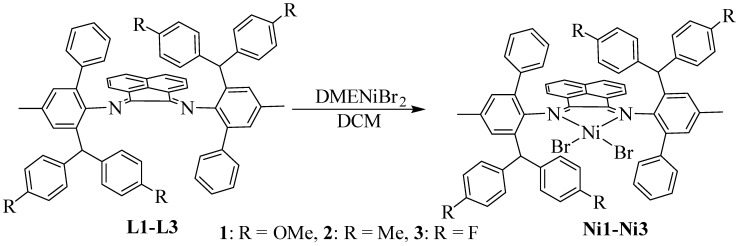
Synthesis of hybrid bulky acenaphthene-based *α*-diimine Ni(II) complexes.

**Figure 1:**
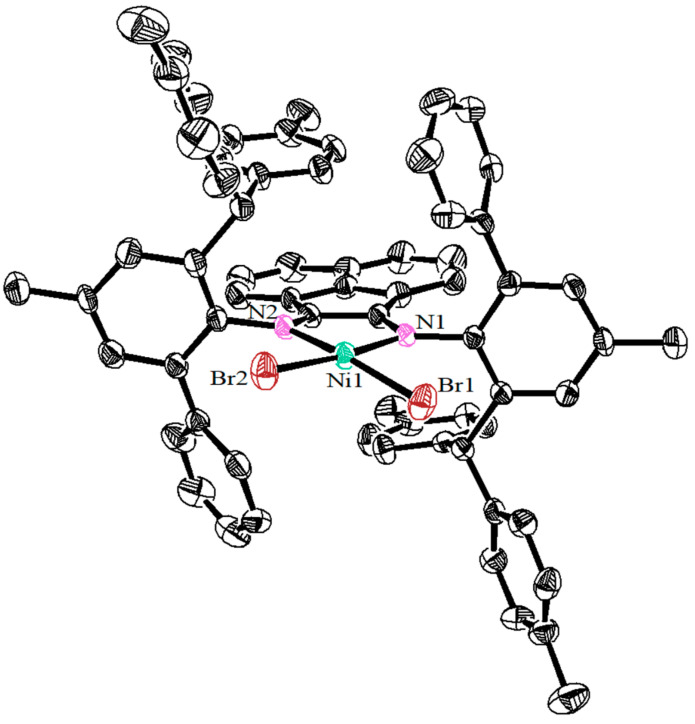
Single crystal structure of **Ni2** (2233726) presented by ORETP (Oak Ridge Thermal Ellipsoid Plot) with 30% probability level, and H atoms were omitted for clarity.

The hybrid bulky acenaphthene-based α-diimine ligands (**L1-L3**) were obtained based on previously reported literature [37]. The target nickel complexes (**Ni1-Ni3**) were yielded by reacting the ligands with equivalent (1,2-dimethoxyethane)nickel dibromide (DMENiBr_2_) in dichloromethane (DCM) at an ambient temperature (Scheme 2). Ideal yields (63–82%) could be achieved and all the nickel complexes were characterized by elemental analysis and Infrared Spectrum (Figures S13–S15). As shown in Figure 1, the single crystal of **Ni2** was fortunately obtained by layering its DCM solution with diethyl ether in the glove box at room temperature. The nickel center adopts a slightly distorted square planar geometry, which is inconsistent with the classical tetrahedral conformation adopted by most previous α-diimine nickel complexes [38,39,40,41,42]. This may be caused by the squeezing of the surrounding bulky *o*-aryl substituents. Moreover, the Ni(II) complex adopts the *anti*-configuration with *ortho*-phenyl groups located on the opposite side.

### 2.2. Ethylene Polymerization

**Figure 2:**
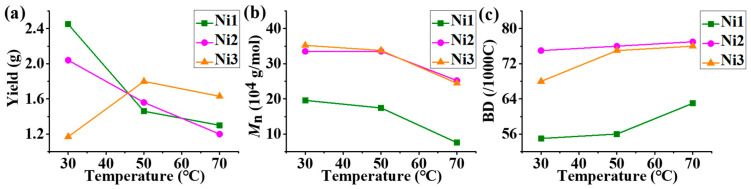
The comparison of polyethylene catalyzed by **Ni1-3** within a certain temperature range (30–70 °C): (**a**) yield; (**b**) molecular weight; (**c**) branching density.

**Figure 3:**
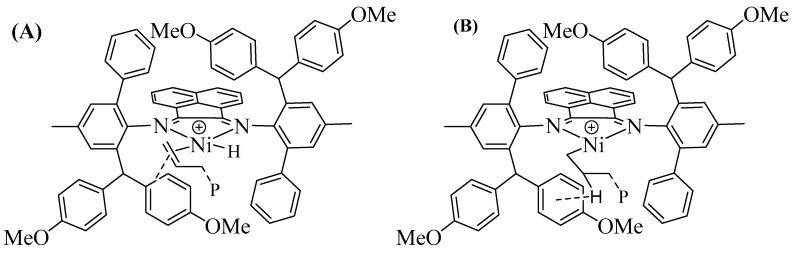
The weak neighbor–group interactions promote the chain transfer (**A**) and suppress β-H elimination (**B**) in ethylene polymerization.

With the activator of 300 eq. Et_2_AlCl, all the nickel complexes exhibited high catalytic activities in ethylene polymerization in the level of 10^6^ g mol^−1^ h^−1^, over a wide temperature range from 30 °C to 70 °C (Table 1). The polymerization activity gradually decreased with raising temperature in most trials, where the highest activity was obtained by **Ni1** at 30 °C (Figure 2a). High-molecular-weight (Figures S10–S12) polyethylene with moderate to high branching density (Figures S1–S3 and S16) were yielded and the molecular weight gradually decreased with raising temperatures in most trials, where the highest molecular weight was obtained by **Ni3** at 30 °C (Figure 2b). The above phenomena could be explained that higher temperatures promote more chain transfer than chain growth in polymerization process. Most interestingly, the polyethylene generated by the methoxy-substituted **Ni1** exhibited significantly lower molecular weights and branching densities than those by the other two complexes. This may be caused by electron-rich aryl-metal weak neighbor–group interactions, which are described in many known reports [43,44,45,46,47]. The weak neighbor–group interactions between *p*-methoxyphenyl and nickel center promote the chain transfer and retard the chain walking by suppressing β-H elimination in ethylene polymerization (Figure 3).

**Figure 4:**
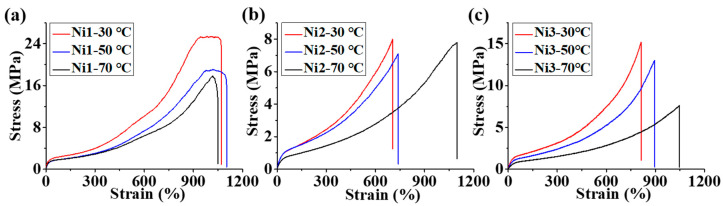
The comparisons of stress–strain curves for polyethylene samples produced by **Ni1-3** at different temperatures (**a**–**c**).

**Figure 5:**
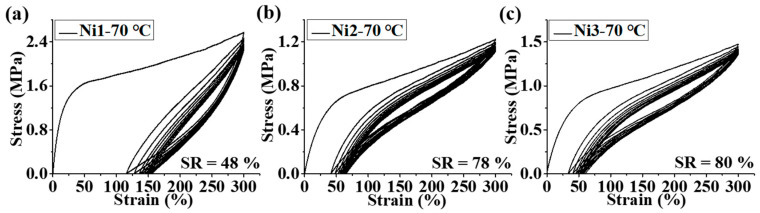
Hysteresis experiment plots of polyethylene samples produced by **Ni1-3** at 70 °C for ten cycles at 300% strain (**a**–**c**).

We further analyzed the mechanical properties of all the branched polyethylenes generated by **Ni1-3**. The polyethylene products generated by **Ni1** showed both high stress (18–25 MPa) and high strain (943–1019%) at break values while those yielded by **Ni2-3** displayed moderate stress (7–15 MPa) and high strain (704–1097%) at break values (Table 2, Figure 4). The lower branching density and a higher melting point (Figures S4–S9) of polyethylene produced by **Ni1** are conducive to having higher tensile strength. Similar phenomena have also been reported in other literature [17,36]. A deeper reason may be that a higher melting point and lower branching density are conducive to increasing the crystallinity of the polymer and thus enhancing its physical crosslinking strength. These polyethylene mechanical parameters are susceptible to variations in polymerization temperature. Typically, polyethylene obtained at high temperatures tends to have a lower Young’s modulus and a higher strain at break values (Table 2, Figure 4). This is primarily because high temperatures facilitate the chain walking of the catalyst and result in higher-branched polyethylene with correspondingly lower polyethylene crystallinity. Hysteresis experiments were carried out to investigate strain recovery (SR) values of the polyethylene samples obtained at 70 °C by **Ni1-3**. As shown in Figure 5, the polyethylene produced by **Ni1** at 70 °C presented a moderate recovery performance (SR = 48%) while those yielded by **Ni2-3** displayed better ones (SR = 78–80%). This is also the result of the higher melting point and lower branching density of the polyethylene obtained by **Ni1** than those by **Ni2-3**. The above results indicate that we can obtain polyethylene TPEs with an excellent performance by **Ni2-3**-catalyzed ethylene polymerization. The ^13^C analysis of the sample from entry 9, Table 1 indicates that the polyethylene contains a variety of branching, with methyl branching dominating and more than 11% of the branching above C_3+_. The presence of significantly more proportional long-chain branching may help improve elastic recovery.

## 3. Conclusions

A series of bulky unsymmetrical acenaphthene-based α-diimine nickel complexes were synthesized and employed for the ethylene polymerization in this study. These complexes all showed high catalytic activity (~10^6^ g mol^−1^ h^−1^) and the obtained polyethylene products possessed high molecular weights (75.6–352.4 kg/mol) and proper branching densities (55–77/1000C). Most interestingly, the polyethylene produced by the methoxy-substituted **Ni1** exhibited much lower molecular weights and branching densities than those by **Ni2-3**. All the branched polyethylenes produced by **Ni1-3** exhibited moderate to high stress (7–25 MPa) as well as high strain (704–1097%) at break values, and moderate to high strain recovery (48–80%) in the tensile tests. Overall, polyethylene TPEs with ideal performance were successfully prepared by **Ni2-3**-catalyzed ethylene polymerization, which has great potential in the automotive industry and photovoltaic film fields.

## 4. Experiments

### 4.1. General Considerations

Unless otherwise stated, all the chemicals were purchased commercially. Polymerization reactions in this work were all performed via standard Schlenk techniques or in a glove box with N_2_ atmosphere. Deuterated solvents were dried and distilled before being used for NMR. A JEOL JNM-ECZ600R 600 spectrometer (JEOL, Tokyo, Japan) or JEOL JNM-ECZ400R 400 spectrometer (JEOL, Tokyo, Japan) was used to get ^1^H and ^13^C NMR spectra at room temperature. The chemical shifts of the ^1^H and ^13^C NMR spectra were referenced to the residual solvent; the coupling constants are in Hz. Elemental analysis was performed by the Analytical Center of Anhui University. X-ray diffractometer (XRD) (Bruker Smart CCD) (Bruker, Billerica, MA, USA) was applied to characterize the crystal structure at 298(2) K with graphite-monochromated Mo K^α^ radiation (λ = 0.71073 Å). Size exclusion chromatography (SEC) was used to determine the samples’ molecular weight and its distribution at 150 °C with a PL 210 equipped with three columns one Shodex AT-803S and two Shodex AT-806MS (Agilent Technologies, Santa Clara, CA, USA). Differential scanning calorimetry (DSC) analysis was carried out on a TA Instruments Q25 (TA Instruments, Newcastle, DE, USA).

### 4.2. Synthesis of Nickel Complexes

Typically, 1 equivalent (DME) NiBr_2_ and 0.2 mmol ligand were fully dissolved in DCM by vigorous stirring overnight. Subsequently, the brown powders were collected after removing the solvent and washed with hexanes (5 mL) for two times. The resultant product was vacuum dried, finally giving the nickel complexes.

**Ni1** (149 mg, 63 %). Anal. Calcd for (C_68_H_56_Br_2_N_2_NiO_4_): C, 69.00; H, 4.77; N, 2.37. Found: C, 69.12; H, 4.67; N, 2.31. IR: C=N (1613, 1646 cm^−1^).
**Ni2** (184 mg, 82 %). Anal. Calcd for (C_68_H_56_Br_2_N_2_Ni): C, 72.94; H, 5.04; N, 2.50. Found: C, 72.87; H, 5.12; N, 2.63. IR: C=N (1630, 1663 cm^−1^).
**Ni3** (166 mg, 73 %). Anal. Calcd for (C_64_H_44_Br_2_F_4_N_2_Ni): C, 67.69; H, 3.91; N, 2.47. Found: C, 67.76; H, 3.87; N, 2.53. IR: C=N (1606–1658 cm^−1^).

### 4.3. Preparation of Polyethylene TPEs by Nickel Complexes

At first, we dried the glass reactor (350 mL) connected with a high gas pressure line in a blast oven at 60 °C, over 24 h. Then, 300 eq. Et_2_AlCl and 40 mL toluene were mixed in the 350 mL flask. 2 μmol Ni catalyst was dissolved in 2 mL DCM and added to the reaction system by injection. Subsequently, the polymerization was carried out with vigorously stirring at a proper temperature and ethylene pressure of 6 atm for 30 min. It is worth noting that all the above procedures were performed in the glove box with N_2_ atmosphere. Finally, the polyethylene products were precipitated in ethanol, followed by vacuum drying at 25 °C for about 24 h.

## Figures and Tables

**Table 1:** Ethylene Polymerization *^a^*.

Entry	Complex	*T* (°C)	Yield (g)	Act. *^b^*	*M*_n_ (10^4^) *^c^*	*M*_w_/*M*_n_*^c^*	*Brs ^d^*	*T_m_^e^*
1	**Ni1**	30	2.45	2.45	19.59	1.82	55	58, 119
2	**Ni1**	50	1.46	1.46	17.45	1.37	56	66, 114
3	**Ni1**	70	1.30	1.30	7.56	2.12	63	48, 119
4	**Ni2**	30	2.04	2.04	33.53	1.86	75	28
5	**Ni2**	50	1.56	1.56	33.54	1.76	76	26
6	**Ni2**	70	1.20	1.20	25.22	1.65	77	23
7	**Ni3**	30	1.17	1.17	35.24	2.00	68	44
8	**Ni3**	50	1.80	1.80	33.79	1.78	75	27
9	**Ni3**	70	1.63	1.63	24.46	1.38	76	22

*^a^* Reaction conditions: Ni complexes = 2 μmol, 300 eq. Et_2_AlCl, toluene = 40 mL, ethylene = 6 atm, polymerization time = 0.5 h, *^b^* Activity is in unit of 10^6^ g mol^−1^ h^−1^, *^c^* Determined by size exclusion chromatography (SEC) in 1,2,4-trichlorobenzene at 150 °C vs. polystyrene standards, *^d^ Brs* = Number of branches per 1000C, as determined by ^1^H NMR spectroscopy, *^e^* Determined by DSC (second heating).

**Table 2:** Mechanical Properties for Different Polyethylene Samples *^a^*.

Ent.	Complex	*T*/°C	Strain at Break (%) *^b^*	Stress at Break (MPa) *^b^*	SR (%) *^c^*
1	**Ni1**	30	943	25	- *^d^*
2	**Ni1**	50	978	19	- *^d^*
3	**Ni1**	70	1019	18	48
4	**Ni2**	30	704	8	- *^d^*
5	**Ni2**	50	738	7	- *^d^*
6	**Ni2**	70	1097	8	78
7	**Ni3**	30	814	15	- *^d^*
8	**Ni3**	50	895	13	- *^d^*
9	**Ni3**	70	1049	8	80

*^a^* Conditions: carried out by Universal Test Machine (UTM2502) with 10 mm/min at 25 °C. *^b^* Strain and stress at break values. *^c^* The strain recovery (SR) values can be calculated by SR = 100 (ε_a_ − ε_r_)/ε_a_, where ε_a_ is the applied strain and ε_r_ is the strain in the cycle at zero load after 10th cycle. *^d^* Not determined.

## Data Availability

The data presented in this study are available on request from the corresponding author.

## Supplementary Materials

The following supporting information can be downloaded at: https://www.mdpi.com/article/10.3390/molecules28052266/s1, Figures S1–S3: ^1^H NMR spectrum of the polymer. Figures S4–S9: DSC of the polymer. Figures S10–S12: GPC of the polymer. Figures S13–S15: IR of the complex. Figure S16: ^13^C NMR spectrum of the polymer. CCDC number of Ni2 is 2233726. The data can be obtained free of charge from the Cambridge Crystallographic Data Centre via www.ccdc.cam.ac.uk/data_request/cif (accessed on 1 January 2023).

## Sample Availability

Samples of the compounds are available from the authors upon reasonable request.
