# Supplementary material for: Direct Synthesis of Polyethylene Thermoplastic Elastomers Using Hybrid Bulky Acenaphthene-Based α-Diimine Ni(II) Catalysts
Source: Molecules. 2023 Feb 28;28(5):2266. doi: 10.3390/molecules28052266 (PMC10005668; doi:10.3390/molecules28052266)
Supplement: Supplementary file 1 [file molecules-28-02266-s001.zip › molecules-2196161-supplementary.pdf]

Supporting Information for:

Direct Synthesis of Polyethylene Thermoplastic Elastomers Using Hybrid Bulky Acenaphthene-Based α-Diimine Ni(II) Catalysts

Hui Wang [1], Weiqing Lu [2,3], Mingmin Zou [3] and Shengyu Dai [2,3,*]

[1] School of Chemistry and Chemical Engineering, Hefei Normal University, Hefei 230061, China
[2] School of Chemical and Environmental Engineering, Anhui Polytechnic University, Wuhu 241000, China
[3] Information Materials and Intelligent Sensing Laboratory of Anhui Province, Institutes of Physical Science and Information Technology, Anhui University, Hefei 230601, China
* Correspondence: daiyu@ustc.edu.cn; Tel.: +86-13955189794

1. Spectra Data

1.1 ^{1}H NMR of Representative Polyethylene.

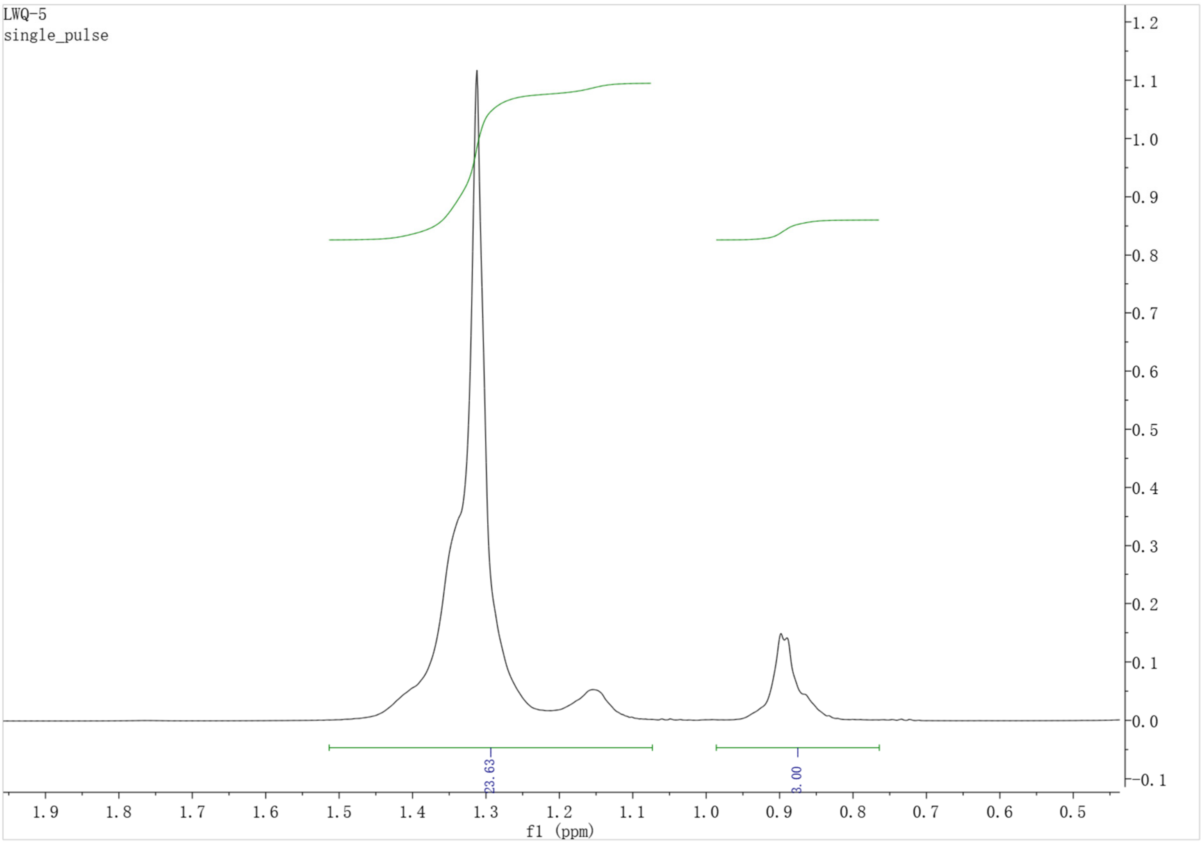

Figure S1. ^{1}H NMR spectrum of the polymer from table 1, entry 4 (d^6-benzene, 70 ºC).

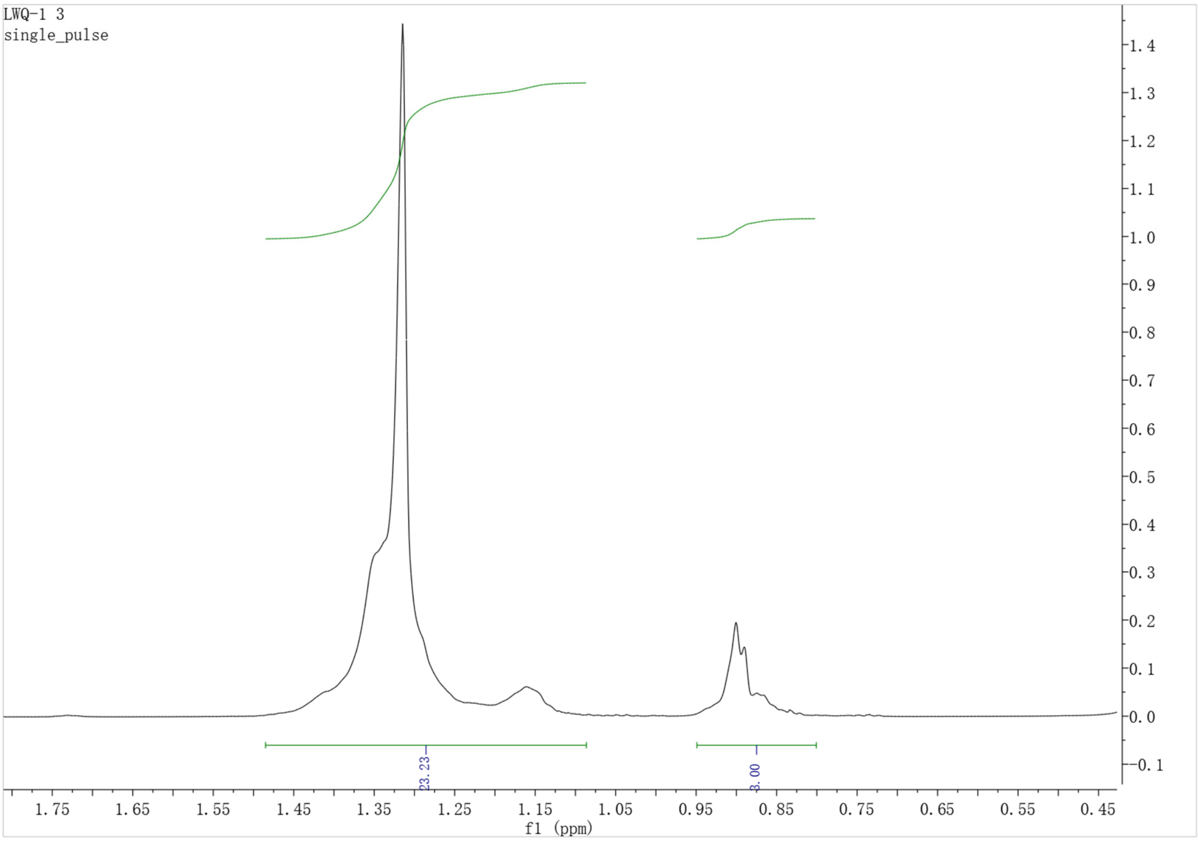

Figure S2. ^{1}H NMR spectrum of the polymer from table 1, entry 5 (d^6-benzene, 70 ºC).

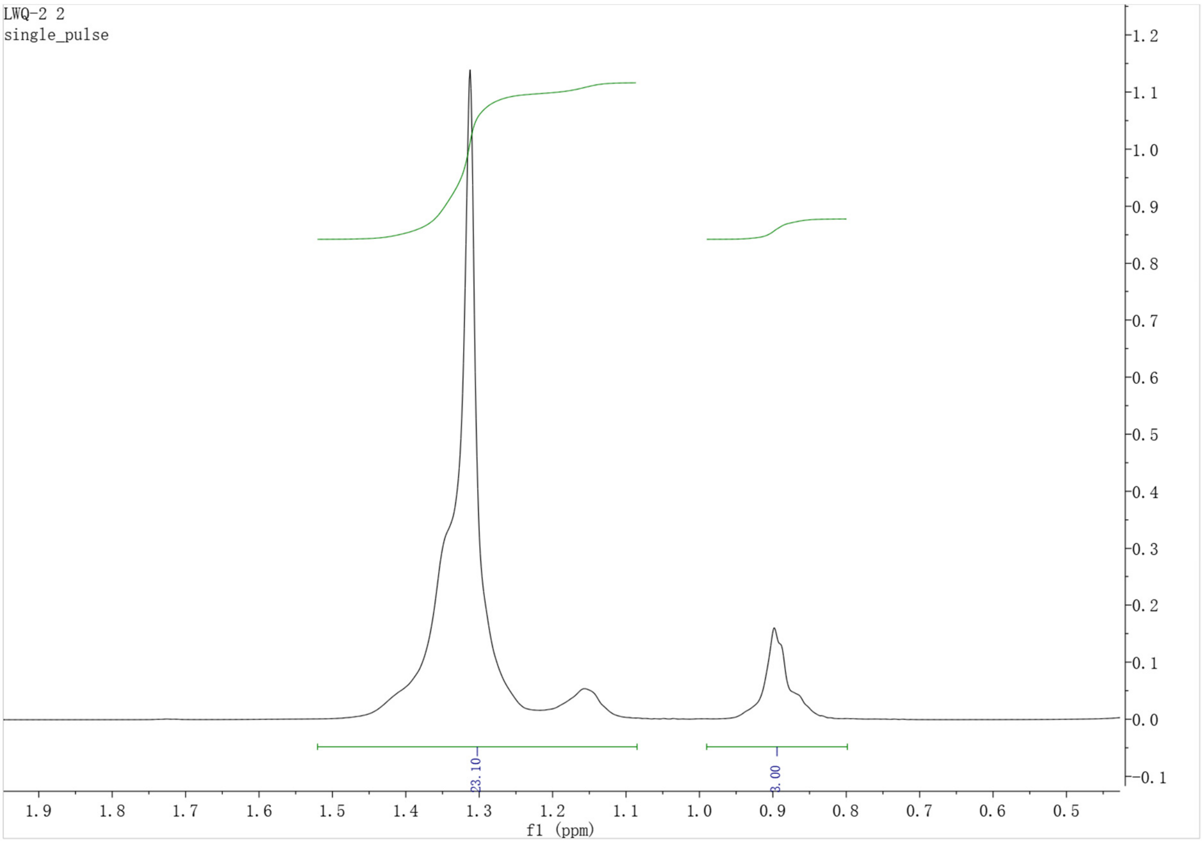

Figure S3. ^{1}H NMR spectrum of the polymer from table 1, entry 6 (d^6-benzene, 70 ºC).

1.2 DSC and GPC of Representative Polyethylenes.

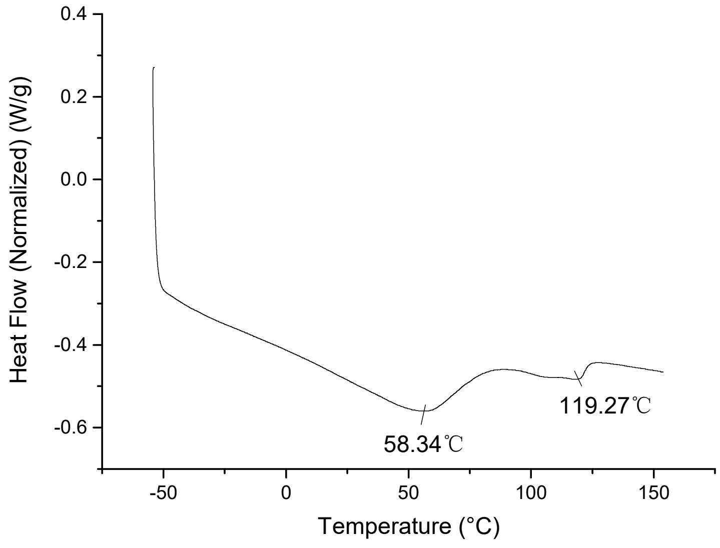

Figure S4. DSC of the polymer from table 1, entry 1.

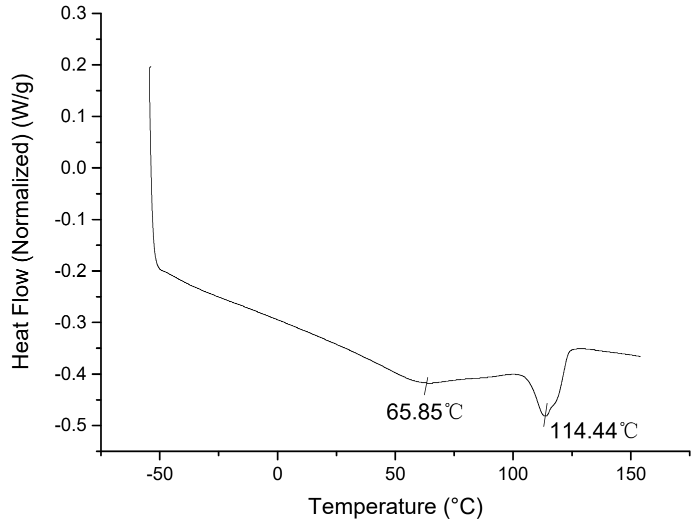

Figure S5. DSC of the polymer from table 1, entry 2.

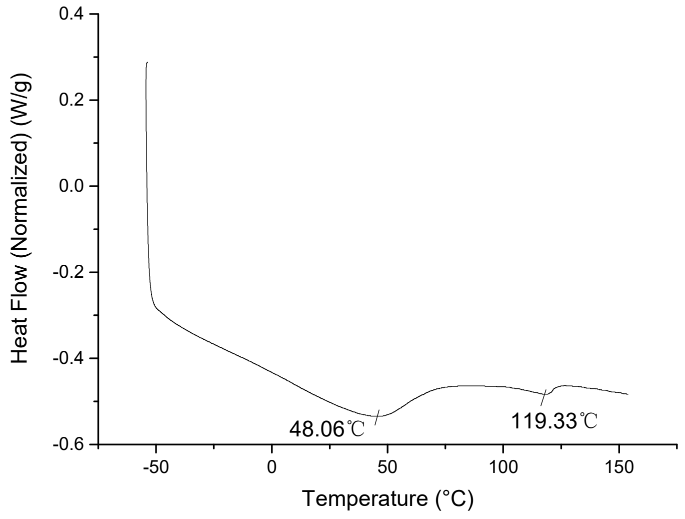

Figure S6. DSC of the polymer from table 1, entry 3.

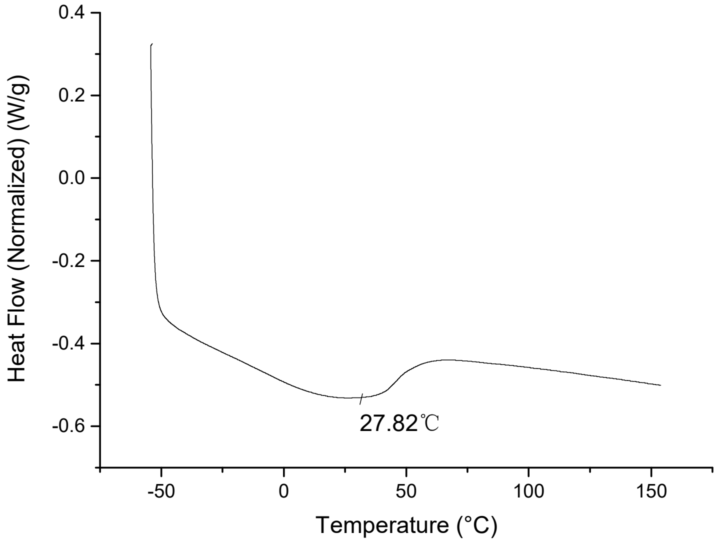

Figure S7. DSC of the polymer from table 1, entry 4.

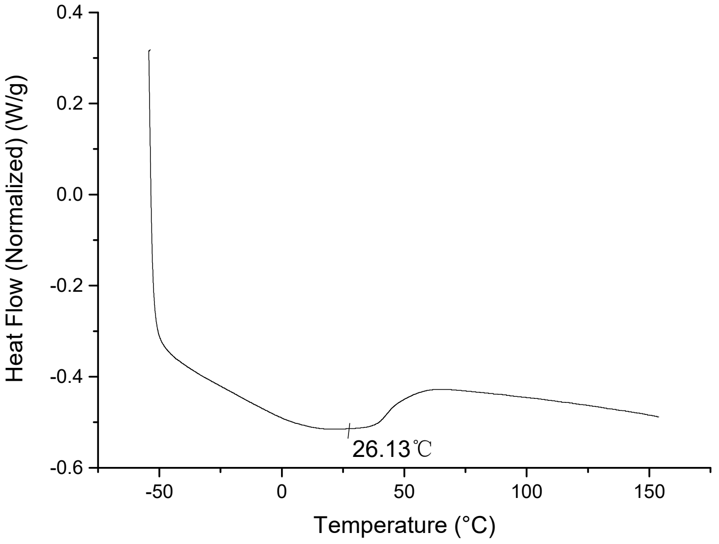

Figure S8. DSC of the polymer from table 1, entry 5.

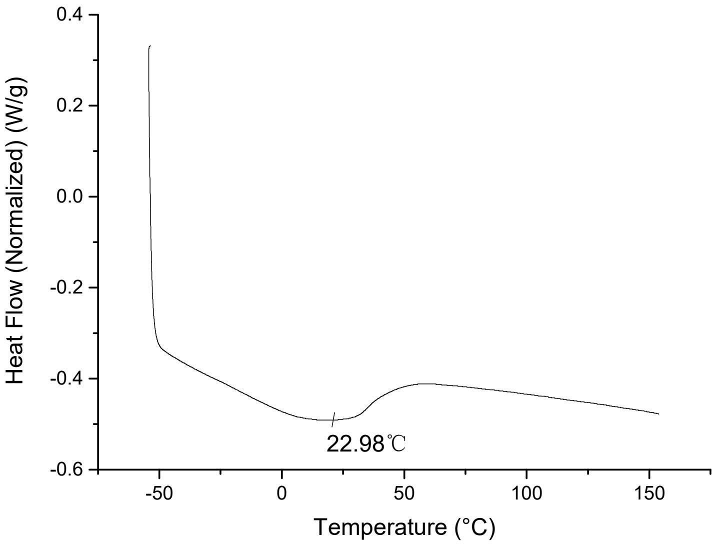

Figure S9. DSC of the polymer from table 1, entry 6.

MW Averages

Mp: 255739	Mn: 195932	Mv: 312451	Mw: 356133
Mz: 1287861	Mz+1: 3893975	PD: 1.8176	

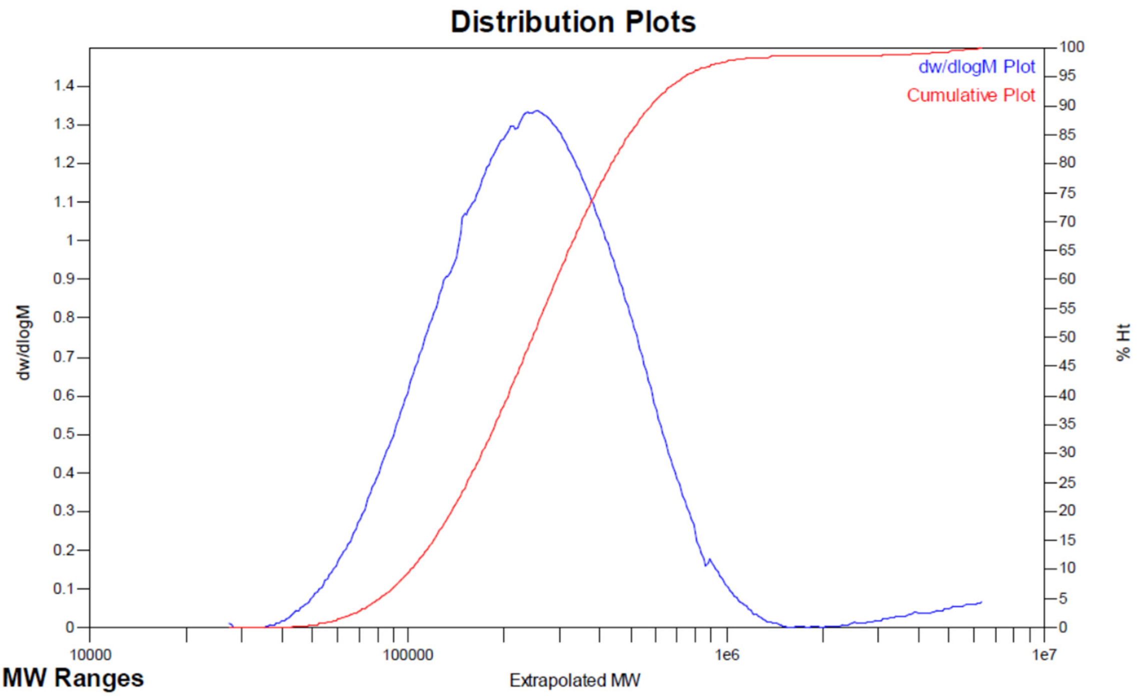

Figure S10. GPC of the polymer from table 1, entry 1.

MW Averages

Mp: 555004	Mn: 335343	Mv: 579452	Mw: 624788
Mz: 1000112	Mz+1: 1422377	PD: 1.8631	

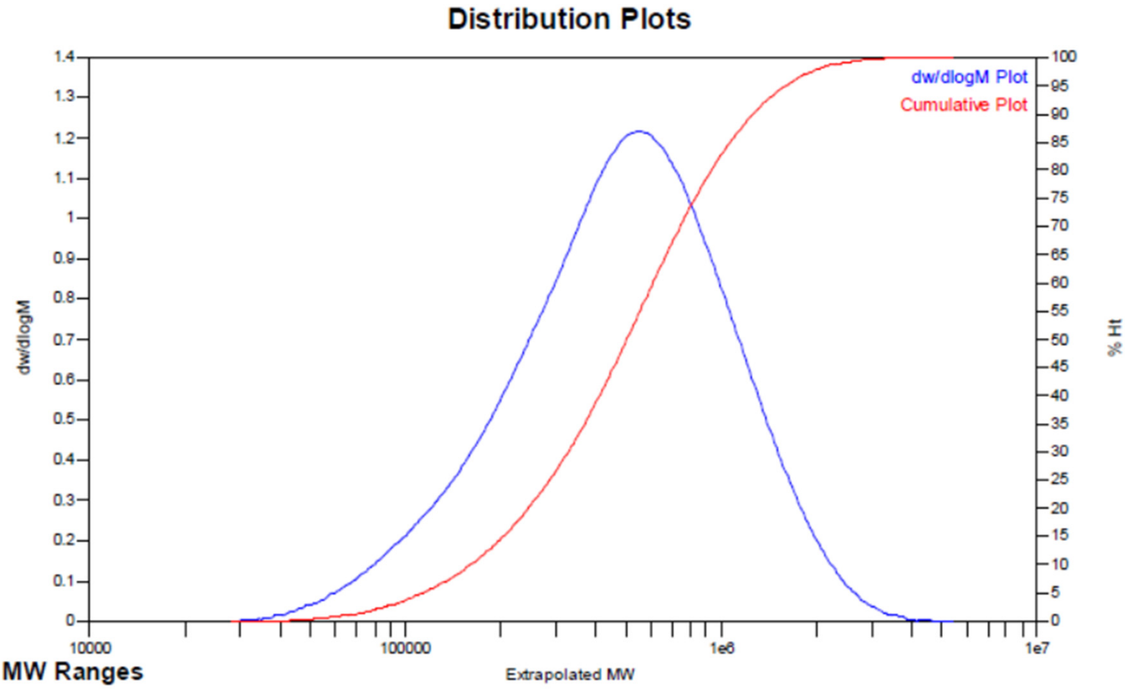

Figure S11. GPC of the polymer from table 1, entry 4.

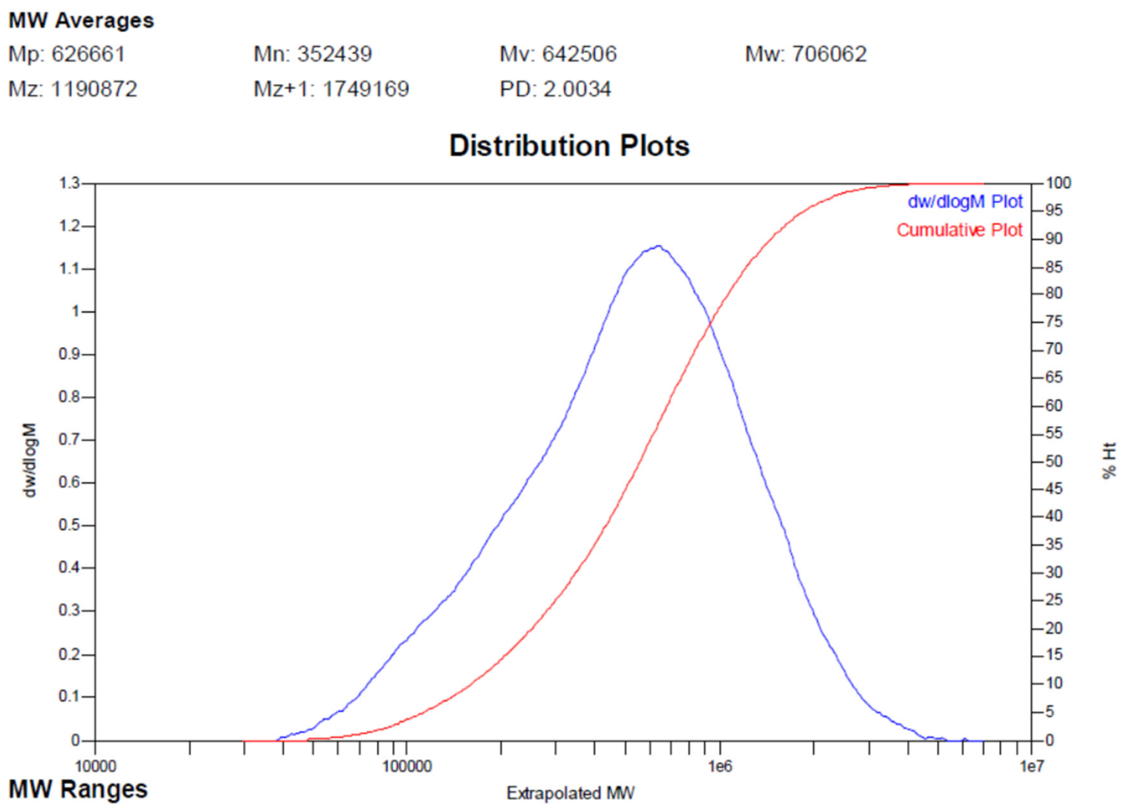

Figure S12. GPC of the polymer from table 1, entry 7.

1.3 Infrared Spectra of the Complexes.

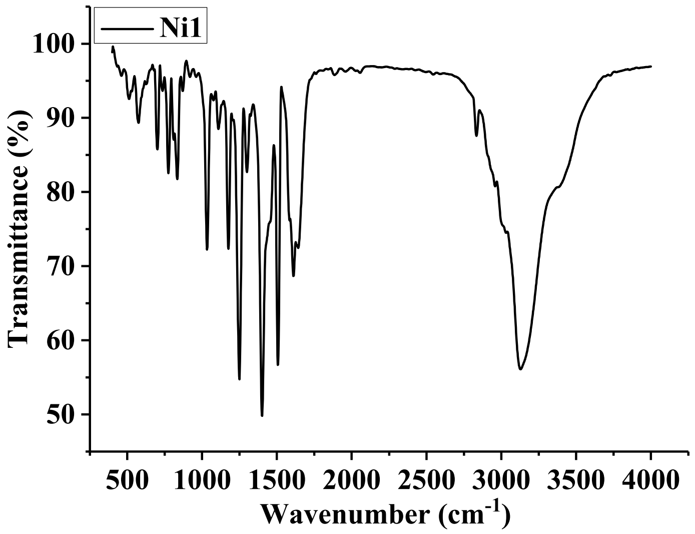

Figure S13. IR of the complex **Ni1**.

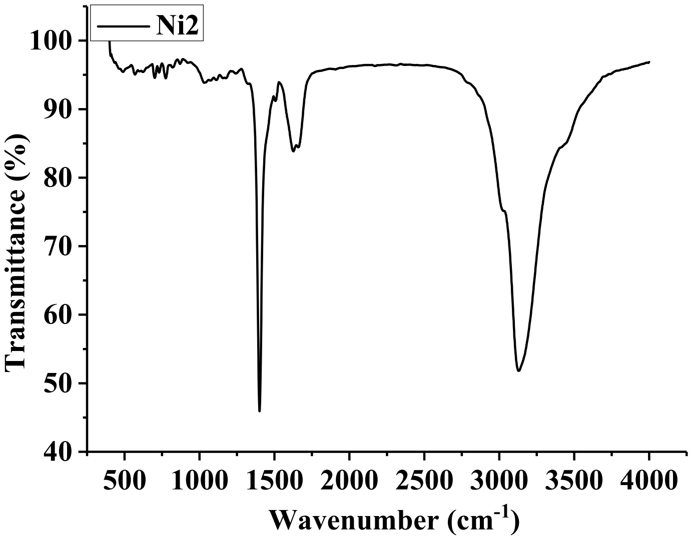

Figure S14. IR of the complex **Ni2**.

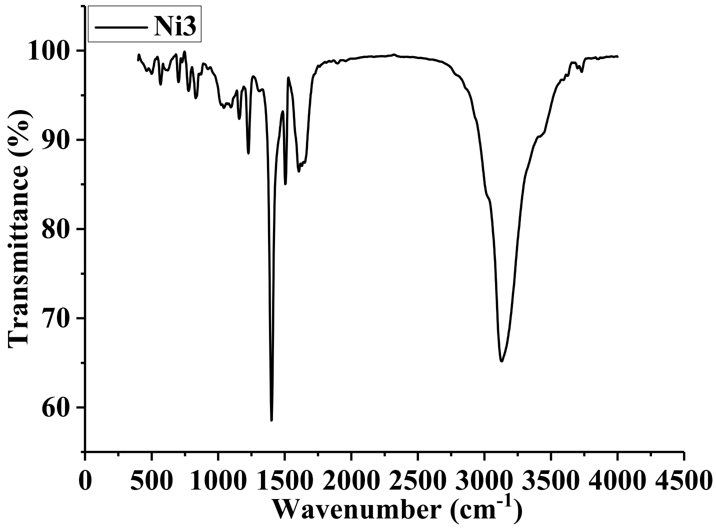

Figure S15. IR of the complex **Ni3**.

1.4 ^{13}C NMR of Representative Polyethylene.

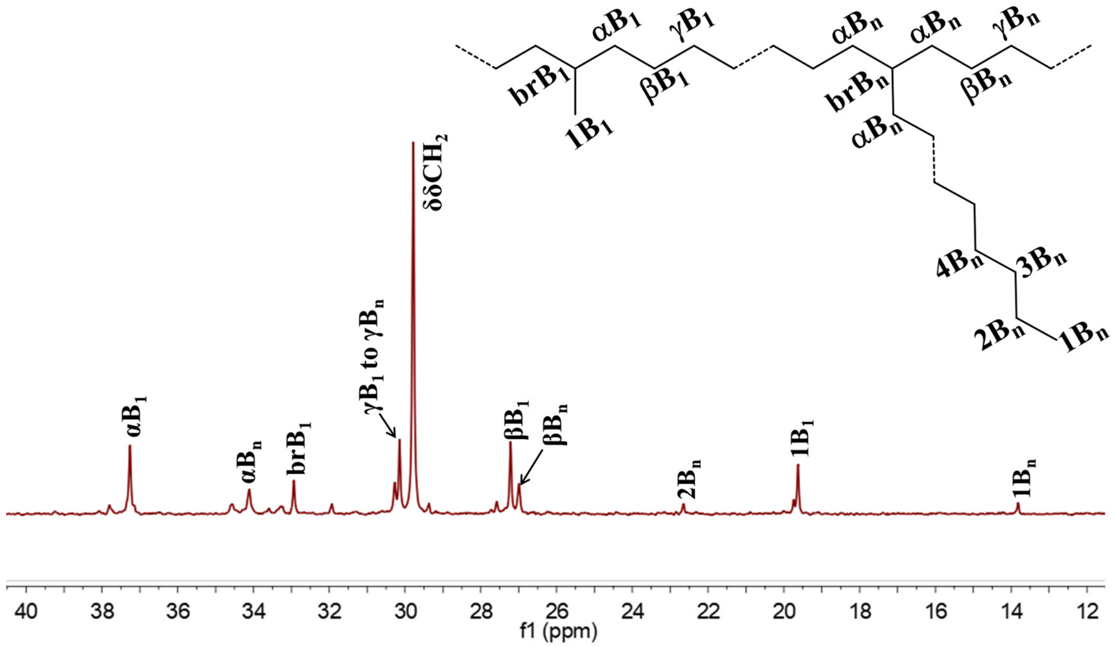

Figure S16. ^{13}C NMR spectrum of the polymer from table 1, entry 7 (d^6-benzene, 70 ºC).
